# Comprehensive genomic profiling identifies novel NTRK fusions in neuroendocrine tumors

**DOI:** 10.18632/oncotarget.26260

**Published:** 2018-11-09

**Authors:** Darren S. Sigal, Munveer S. Bhangoo, Jonathan A. Hermel, Dean C. Pavlick, Garrett Frampton, Vincent A. Miller, Jeffrey S. Ross, Siraj M. Ali

**Affiliations:** ^1^ Division of Hematology/Oncology, Scripps Clinic Medical Group, La Jolla, CA, USA; ^2^ Department of Graduate Medical Education, Tulane University School of Medicine, New Orleans, LA, USA; ^3^ Foundation Medicine, Inc. Cambridge, MA, USA

**Keywords:** next-generation sequencing, neuroendocrine tumor, NET, NTRK, neuroendocrine cancer

## Abstract

CGP results from >60,000 cases were screened to identify NTRK fusion events from cases of neuroendocrine tumors. 2417 NET patients from diverse anatomic sites were identified. From this dataset, six cases harbored NTRK fusions which included intra- and inter-chromosomal translocations. A NTRK fusion frequency of approximately 0.3% was found across all subtypes of NETs. Three cases involved translocations of NTRK1 with unique fusion partners (GPATCH4, PIP5K1A, CCDC19). Co-occurring alterations occurred in five cases. NTRK alterations were identified in nearly the full spectrum of NETs, including from the small intestine, pancreas, lung, and others. With the late stage clinical development of NTRK TKIs (including entrectinib and larotrectinib), these findings may further inform targeted approaches to therapy in NET.

## INTRODUCTION

We recently reported on a patient with the first identified *NTRK* fusion (*ETV6:NTRK3)* in a neuroendocrine tumor (NET) [1]. *NTRK1, 2,* and *3* encode the neurotrophic tropomyosin receptor kinase (TRK) family of receptor tyrosine kinases, TRKA, TRKB, and TRKC, respectively, and *NTRK* alterations are known to be oncogenic [2]. This patient was accrued to the STARTRK2 trial (NCT02568267) and experienced a dramatic and protracted response to entrectinib, an oral tyrosine kinase inhibitor (TKI) of the protein products of *NTRK, ROS1*, and *ALK* alterations. This response suggested that *NTRK* fusions may play an important role in NET pathogenesis made even more significant by the advent of *NTRK* targeting therapies. Next-generation sequencing (NGS) studies of NETs of the small intestine, pancreas, and lung had not previously revealed *NTRK* fusions in NETs [3–5]. We sought to interrogate a large NET database assayed with comprehensive genomic profiling (CGP) to document additional *NTRK* fusions in NET.

## RESULTS

CGP was performed on specimens from 2417 NET patients from diverse anatomic sites in the course of clinical care. From this dataset, six cases harbored *NTRK* fusions which included intra- and inter-chromosomal translocations (see Table 1). Of these cases, five were females. The anatomic sites of origin included pancreas (n=2), uterus (1), lung (1), and unknown (2). Three cases involved translocations of *NTRK1* with unique fusion partners (*GPATCH4*, *PIP5K1A*, *CCDC19*). Three cases had the *NTRK* fragment in the 5’ position to its fusion partner. Fusions involving *NTRK2* and *NTRK3* were identified in one and two cases, respectively. Co-occurring alterations occurred in five cases. Of non-*NTRK* genes altered, *TP53* was the most common occurring in 50% (3/6) cases. Additional co-occurring alterations included *ARID1A, ATM, CDKN2A, EPHA3, MYC, NFE, PTEN, RB1, SLIT2*, and *SPTA1*. Two cases had an alteration involving the MAPK pathway (*KRAS* G12D and *KRAS Q61R)*; there were no other alterations involving *RAF, MEK*, or *ERK*. The mean tumor mutational burden (TMB) was 3.81 mutations per DNA megabase (range 0.87-7.2 mut/Mb).

**Table 1:** Results of CGP analysis demonstrating six *NTRK* translocations occurring in neuroendocrine tumors across multiple anatomic sites of origin

Tumor Type	Sex	NTRK Fusion Product	Rearrangement Type	Supporting Reads	In-Strand?	In-Frame?	TMB (Mut/Mbp)	Co-Occurring Alterations
Lung large cell neuroendocrine carcinoma	female	NTRK3:intergenic region	Duplication	17	Yes	N/A	7.2	PTEN P95L, RB1 R467^*^, TP53 splice site 375+1G>T
Primary undifferentiated neuroendocrine carcinoma	male	PIP5K1A:NTRK1	Deletion	20	Yes	No	0.87	KRAS Q61R, NFE2L2 D61G
Pancreas neuroendocrine carcinoma	female	NTRK1:CCDC19	Duplication	29	No	N/A	3.48	CDKN2A R58^*^, KRAS G12D, TP53 R175H, SPTA1 truncation intron 51
Uterus neuroendocrine carcinoma	female	NTRK1:GPATCH4	Duplication	252	No	N/A	4.35	ARID1A Q1334_R1335insQ, ATM L243S, TP53 T284fs^*^61, LRP1B loss of exons 4-19, EPHA3 amplification, MYC amplification, RB1 duplication of exon 3-12
Pancreas neuroendocrine carcinoma	female	ETV6:NTRK3	Translocation	167	Yes	Yes	6.09	SLIT2 splice site 2346-56_2346-2del55
Primary undifferentiated neuroendocrine tumor	female	SQSTM1:NTRK2	Translocation	6	Yes	Yes	0.87	None

## DISCUSSION

Our analysis identified six NET specimens with *NTRK* fusions out of a total of 2417 evaluated in the Foundation Medicine database. Including the index patient from the case report, we found a *NTRK* fusion frequency of approximately 0.3% across all subtypes of NETs. *NTRK* alterations were identified in nearly the full spectrum of NETs, including from the small intestine, pancreas, lung, and others. These gene fusions were diverse with *NTRK1, 2, 3* fragments each attached to a unique fusion partner. Two patients had a *NTRK1* fusion with co-occurring mutations in *KRAS Q61R* and *KRAS G12D*, respectively. Although it is unusual for *KRAS* mutations to co-occur with other driver tyrosine kinase alterations, this scenario has been reported with TKI efficacy despite the *KRAS* mutation [6]. In three patients, *NTRK* was 5’ to its fusion partner. Oncogenic *NTRK* fusions generally occupy the 3’ fusion position suggesting that the three 5’ *NTRK* fusions we report may not be functional. However, a variety of alternative oncogenic *NTRK* alterations have been reported, including point mutations, deletions, duplications, and other less well-described mechanisms [7–10]. In addition, the fusions we report satisfy Foundation Medicine's reporting rules and therefore would have qualified for the STARTRK2 entrectinib registrational trial.

No previous NGS analyses of NETs have identified *NTRK* gene fusions. We identified *NTRK* gene fusions in two pancreas NET patients despite the absence of these gene fusions among 102 pancreas NET patients screened with whole genome and RNA sequencing [4]. The Foundation Medicine analysis utilized targeted exome sequencing deploying intron baiting for all coding exons of *NTRK1,2,3* with additional baits for introns 7-11 and 13 of *NTRK1* and intron 12 of *NTRK2*. For a tumor fraction specimen of >20%, intron baiting was reported to have a sensitivity of 100% and a positive predictive value of >98% [11]. In contrast, whole genome and RNA sequencing have been preferred methods for detecting translocations that are large and have numerous upstream fusion partners, similar to *NTRK* translocations. The most likely explanations for the discrepancy of *NTRK* fusion detection among these reports is the low absolute frequency of *NTRK* fusions and the relatively small number of pancreatic NETs that were screened in the Scarpa, et al paper.

*NTRK* fusions have been detected at a low frequency in a variety of cancers, but appear to have a higher prevalence in rare cancers. Analysis of the RNA-seq data set from The Cancer Genome Atlas detected *NTRK* fusions in only nine of 20 solid tumor types screened. In these nine tumor types the *NTRK* fusion prevalence ranged from 0.09% to 2.4% (Table 2) [12]. Our finding of 0.3% *NTRK* fusion rate in NETs indicates that these fusions are relatively common in NETs compared to many other malignancies.

**Table 2:** Frequency of NTRK fusion products across multiple tumor types

Tumor Type	No. of Tumors harboring NTRK fusion product/Total No. Samples Tested	Percent (%)
Thyroid carcinoma	12/498	2.41
Sarcoma	1/103	0.97
Colon adenocarcinoma	2/286	0.70
Glioblastoma multiforme	1/157	0.64
Head and neck squamous cell carcinoma	2/411	0.49
Brain low-grade glioma	2/461	0.43
Skin cutaneous melanoma	1/374	0.27
Lund adenocarcinoma	1/513	0.19
Breast invasive carcinoma	1/1072	0.09

This analysis has several limitations. We are missing information on stage, grade, Ki-67 status, and NET subtypes screened. Our report also lacks orthogonal validation to ensure the *NTRK* fusions we report are in-frame and functional. Foundation Medicine does not store tissue for this purpose so these studies were simply not possible. However, DNA based testing is well established for detecting clinically actionable *NTRK* fusions and is allowed for accrual to *NTRK* inhibitor trials.

Our report of a 0.3% prevalence rate is on par with genomic alterations that have impacted standard of care in other malignancies. Malignancies are increasingly defined by even ultra-rare genomic events. Late stage clinical development of entrectinib and larotrectinib, TKIs with high affinity binding for *NTRK* fusion protein products that have reported remarkable response and survival endpoints in various basket studies, makes the finding of *NTRK* fusions throughout the spectrum of NET subtypes clinically important [13, 14]. Although additional efforts can further clarify the prevalence of *NTRK* fusions in NET, determination of *NTRK* fusion status should be incorporated into the care of NET patients.

## MATERIALS AND METHODS

The methods used for genomic profiling have been previously described [15, 16]. Formalin-fixed, paraffin-embedded slides or blocks from tumor samples were submitted to a Clinical Laboratory Improvement Amendments (CLIA)-certified, College of American Pathologists-accredited reference laboratory (Foundation Medicine, Cambridge, MA). Tumor samples submitted for profiling were reviewed by board-certified pathologists for tumor purity as well as the diagnosis made by the treating physicians. At least 50 ng of DNA per specimen was extracted. Next-generation sequencing was performed on hybridization-captured, adaptor ligation–based libraries to high, uniform coverage (> 500×) for all coding exons of 315 cancer-related genes and 28 genes commonly rearranged in cancer. Base substitutions, short insertions, deletions, copy number changes, gene fusions, and rearrangements were identified and reported for each patient sample. CGP results from >60,000 cases were reviewed from the Foundation Medicine database. NTRK fusion events from cases of neuroendocrine tumors were identified and reported as below.
